# Impact of Intermittent Fasting on Lipid Profile–A Quasi-Randomized Clinical Trial

**DOI:** 10.3389/fnut.2020.596787

**Published:** 2021-02-01

**Authors:** Naseer Ahmed, Javeria Farooq, Hasan Salman Siddiqi, Sultan Ayoub Meo, Bibi Kulsoom, Abid H. Laghari, Humaira Jamshed, Farooq Pasha

**Affiliations:** ^1^Department of Biological and Biomedical Sciences, Aga Khan University, Karachi, Pakistan; ^2^Department of Physiology, College of Medicine, King Saud University, Riyadh, Saudi Arabia; ^3^Postgraduate Programme-Training and Monitoring, Bahria University Medical and Dental College, Karachi, Pakistan; ^4^Department of Medicine, Section of Cardiology, Aga Khan University, Karachi, Pakistan; ^5^Integrated Sciences and Mathematics, Dhanani School of Science and Engineering, Habib University, Karachi, Pakistan; ^6^Economics, Boston College, Chestnut Hill, MA, United States

**Keywords:** intermittent fasting, lipid profile, weight reduction, cardioprotection, healthy life style

## Abstract

**Background:** Sub-optimal HDL is a prognostic marker of cardiovascular disease. South Asia has a high prevalence of sub-optimal HDL compared to other parts of the world. Intermittent fasting (IF) is a type of energy restriction which may improve serum HDL and other lipids thereby reducing the risk of cardiovascular diseases.

**Objective:** The aim of the study was to evaluate the effect of IF on lipid profile and HDL-cholesterol in a sample of South Asian adults.

**Methods:** A 6-week quasi-experimental (non-randomized) clinical trial was conducted on participants with low HDL (< 40 mg/dl for men and < 50 mg/dl for women). Participants of the control group were recommended not to change their diet. The intervention group was recommended to fast for ~12 h during day time, three times per week for 6 weeks. Pulse rate, blood pressure, body weight, waist circumference, serum lipid profile, and blood glucose levels were measured at baseline and after 6 weeks.

**Result:** A total of 40 participants were enrolled in the study (*N* = 20 in each group), while 35 (20 control and 15 intervention) completed the trial and were included in data analysis of the study. Body measurements, including body weight, BMI and waist circumference, showed significant interaction effects (*p*'s < 0.001), indicating that there were larger reductions in the IF group than in the control group. Significant interaction effects were also observed for total (*p* = 0.033), HDL (*p* = 0.0001), and LDL cholesterol (*p* = 0.010) with larger improvements in the IF group.

**Conclusion:** This study suggests that intermittent fasting may protect cardiovascular health by improving the lipid profile and raising the sub-optimal HDL. Intermittent fasting may be adopted as a lifestyle intervention for the prevention, management and treatment of cardiovascular disorders.

**Clinical Trial Registration:** NCT03805776, registered on January 16, 2019, https://clinicaltrials.gov/ct2/show/NCT03805776

## Introduction

It is well-documented that dyslipidemia, characterized by high concentration of serum total cholesterol (TC), low-density lipoprotein cholesterol (LDL-C) and triglycerides (TG) with low levels of high-density lipoprotein cholesterol (HDL-C), is linked to cardiovascular disease (CVD) (1). Some studies have shown that low HDL-C, with normal LDL-C and triglyceride levels can be as dangerous for coronary health as high LDL-C (2, 3). HDL-C reverses cholesterol transport and reduces the atherosclerotic burden. HDL-C also has anti-inflammatory, anti-oxidative, anti-thrombotic, anti-apoptotic, and vasodilatory properties (4). Various alternative ways for managing dyslipidemia include life style modification, regular exercise and moderate alcohol consumption (5).

Intermittent fasting (IF) can be adopted as a life style modification for good health and balanced lipid profile. IF is type of energy restricted feeding protocol known since long from religious and cultural backgrounds. IF has been extensively studied in animal models. Such studies indicate that IF improves lipid profile (6), protects the heart from ischemic injury, and attenuates post-MI cardiac remodeling (7). Various scientific studies have been conducted on humans to identify the role of different IF methods including alternate day fasting, caloric restriction, Ramadan fasting and periodic fasting etc. Alternate-day fasting (ADF) reduces body weight by 3–7% over 2–3 month, and improves lipid profiles and blood pressure. It was suggested that fasting positively impacts metabolic biomarkers and cardiovascular health while long term effects should be explored (8). A clinical trial of ADF in adults with obesity found it as an effective method for weight reduction and reduction in coronary artery disease risks (9). Additionally, one clinical trial found ADF effective for weight reduction in people with normal and overweight (10). Combination of alternate day fasting with physical activity reports greater changes in body composition and plasma lipid profile and reduces cardiovascular risk as compared to individual treatments (11). Keogh et al. found that IF is as effective for weight management as continuous calorie restriction for 8 weeks (12). The reduced caloric intake and weight loss might explain the effects of IF on the lipid profile which may be translated to improvements in cardio metabolic health (13).

Ramadan fasting studies have shown mixed effects on health. Some studies found reduction in body weight (14) while others report minimal change (15). Similar inconsistencies are reported for the lipid profile and blood glucose levels as well. One explanation could be the confounding variables such as the fasting duration, medications, dietary habits, cultural norms and physical activity (16). Other factors may include methodological differences, seasonal changes, geographical location, daylight exposures etc.

The current study trial was designed to investigate the effects of IF on lipid profile in adults. It was hypothesized that IF will improve the lipid profile and might prevent cardiovascular diseases. The study protocol was different from other previously studied IF methods as it required day time 12 h fasting for 3 days a week for 6 weeks. It had similarity with Ramadan fasting in that the fast was kept from sunrise to sunset but it was different from Ramadan fasting in the aspect that Ramadan fasting requires daily fasting for four continuous weeks. In this study, IF was defined as fasting for 3 days in a week for 6 weeks.

## Methods

### Study Design

This was a quasi-experimental clinical study conducted on employees of the Aga Khan University Hospital. People were informed through emails, phone calls and personal contacts. The Declaration of Helsinki and Good Clinical Practice guidelines was followed. After explaining the study protocol to the participants, written informed consent was collected. Participants did not receive any incentive, monetary or otherwise, for participating in this study. Sample size was calculated by reviewing the previous intermittent fasting trial sample sizes (17, 18). The power of the study for significant improvement in HDL cholesterol was 80%, with a significance level of 5%.

### Participants

Inclusion criteria included age of 20–70 years, with serum HDL <40 mg/dl for men and <50 mg/dl for women. Pregnant women and individuals with self-reported cardiovascular diseases or any other co-morbidity were excluded. Screening was performed and lipid profile was conducted to confirm HDL levels. A total of 40 subjects (20 in each group) were enrolled in the study.

### Data Collection

The employees who agreed to participate in the trial were called for screening. They were asked to bring their lipid profile result from the last 4 weeks, if available. The individuals without such previous lipid profile reports were asked to come after 10–12 h of fasting so that a lipid profile test could be performed. Individuals with low HDL levels indicated either by the previous reports or by currently performed screening lipid profiles were enrolled in the study. Screening and enrolment were completed in 3–4 weeks. Then enrolled participants were invited to a designated room in the Multidisciplinary laboratory of Aga Khan University where questionnaires regarding participants' eating routine and physical activity of participant were completed. Body weight, waist circumference, height and blood pressure were measured. Body fat and water content were measured by an impedance scale. Blood was collected for lipid profile testing and glucose estimation. Participants were called again after 6-weeks whereby the same body parameters were measured and fasting blood was collected.

### Intervention and Control

The participants were distributed into two groups according to their group preference; Control and Intervention. Informed consent form was signed by all the participants. Intervention group was advised to fast for ~12 h during day time (6 A.M.−6 P.M.) for only 3 days/ week for 6 weeks. The intervention group was instructed to take their routine diet in the non-fasting period. The control group continued their usual dietary pattern and were advised to make no changes in lifestyle. Compliance was monitored through phone calls and messages every week for 6 weeks. Although there are no reported adverse effects of intermittent fasting, the contact number of a doctor was given to participants in case of any emergency or concern.

### Ethical Consideration

The clinical trial was approved by Ethics Review Committee of Aga Khan University Hospital with registration number ERC # 2019-0633-2318. The trial was registered at NIH, US National Library of Medicine as NCT03805776. The study protocol was explained in detail to all the participants. Privacy and confidentiality was maintained.

### Sample Analysis

Blood samples were centrifuged on the day of collection at 2500 RPM for 15 min at 4°C. Serum was separated in aliquots and was stored at −20°C for lipid analysis. For performing lipid profile test, cobas c 111 kits (Roche diagnostics, made in Germany) was used with cobas c 111 automated analyzer (Roche Cobas).

### Data Analysis

Data were analyzed using IBM SPSS Statistics 20 and GraphPad Prism 8. Data are presented as mean ± standard deviation (SD) in Tables 1, 2 and figures. However, in Table 3 data is presented as mean difference ± standard error mean (SEM). The level of significance was set to α < 0.05 for all performed two-sided tests. To detect changes over time and respective differences between the groups, a repeated-measures ANOVA (rmANOVA) with factors time (pre, post) × group (IF, Control) was performed to test for interaction effects. In the case of significant interaction effects from the rmANOVA, Bonferroni corrected Student's *t*-tests were calculated for any pre to post differences. For metabolic risk factors, data have been adjusted with mean of body weight of the entire sample at baseline.

**Table 1:** Baseline description of participants.

**Parameters**	**Control**	**Intermittent fasting**	**Intermittent fasting**
		**Enrolled** ** (*n* = 20)**	**Completed trial (*n* = 15)**
Age (year)	42.30 ± 13.50	36.05 ± 12.06	37.80 ± 12.25
**Gender**			
Male	12	13	8
Female	8	7	7
**BMI (kg/m**^**2**^**)**			
Underweight	1	1	1
Normal	8	6	2
Overweight	10	11	10
Obese	1	2	2
**Blood Pressure (mmHg)**			
Systolic	120.90 ± 15.41	115.11 ± 13.69	114.93 ± 13.86
Diastolic	80.65 ± 8.18	80.39 ± 6.71	79.80 ± 5.73
**Health condition (ratio)**	Yes: No	Yes: No	Yes: No
Dyslipidemia	20:00	20:00	15:00
Hypertension	3:17	0:20	0:15
Diabetes mellitus	0:20	0:20	0:15
Cigarette smoking	2:18	0:20	0:15
Family history of IHD/CVD	2:18	3:17	3:12

**Table 2:** Changes in parameters before (baseline) and after (post) intermittent fasting with Control (*n* = 20) and IF (*n* = 15).

**Parameters**	**Groups**	**Baseline**	**Post**	**Interaction effect**	**Time effect**
				**(Time x group)**	
Body weight (kg)	Control	73.07 ± 11.63	73.03 ± 11.56	0.0001^**^	0.0001^**^
	IF	75.73 ± 12.78	72.63 ± 12.23		
BMI (kg/m^2^)	Control	25.67 ± 3.58	25.63 ± 3.50	0.0001^**^	0.0001^**^
	IF	27.62 ± 4.14	26.64 ± 4.01		
Waist circumference(cm)^∧^	Control	34.57 ± 4.39	34.44 ± 4.46	0.001^*^	0.111
	IF	34.73 ± 4.74	33.77 ± 4.75		
Body fat (%)	Control	31.29 ± 8.02	31.40 ± 7.94	0.255	0.456
	IF	31.87 ± 6.14	31.36 ± 7.17		
Total cholesterol (mg/dl)^∧^	Control	185.00 ± 36.27	182.40 ± 34.48	0.033^*^	0.859
	IF	198.29 ± 38.82	182.09 ± 30.56		
Triglycerides (mg/dl)^∧^	Control	138.70 ± 78.30	137.00 ± 74.57	0.075	0.662
	IF	135.60 ± 60.13	122.80 ± 61.48		
LDL (mg/dl)^∧^	Control	102.32 ± 26.27	104.69 ± 24.42	0.010^*^	0.333
	IF	111.04 ± 40.71	105.95 ± 33.04		
HDL (mg/dl)^∧^	Control	34.45 ± 6.81	34.01 ± 6.48	0.0001^**^	0.055
	IF	35.60 ± 6.45	38.62 ± 6.45		
Blood glucose (mg/dl)^∧^	Control	82.35 ± 11.90	81.83 ± 7.69	0.623	0.339
	IF	80.40 ± 10.78	80.87 ± 10.04		

*Results are presented as mean ± SD. IF, intermittent fasting group*;

* *p < 0.05*,

** p < 0.001; parameters with

∧ *symbol represents adjustment with baseline body weight of the entire sample*.

**Table 3:** Mean difference in parameters after (post) and before (baseline) intermittent fasting with Control (*n* = 20) and IF (*n* = 15).

**Parameters**	**Groups**	**Mean difference**	***P*-value**
Body weight (kg)	Control	−0.05 ± 0.16	0.757
	IF	−3.10 ± 0.19	0.0001^**^
BMI (kg/m^2^)	Control	−0.04 ± 0.11	0.725
	IF	−0.98 ± 0.13	0.0001^**^
Waist circumference(cm)^∧^	Control	−0.11 ± 0.16	0.480
	IF	−0.98 ± 0.18	0.0001^**^
Body Fat (%)	Control	0.11 ± 0.35	0.760
	IF	−0.51 ± 0.40	0.215
Total cholesterol (mg/dl)^∧^	Control	−2.69 ± 3.92	0.498
	IF	−16.08 ± 4.53	0.001^*^
Triglycerides (mg/dl)^∧^	Control	−1.68 ± 3.95	0.673
	IF	−12.82 ± 4.57	0.008^*^
LDL (mg/dl)^∧^	Control	2.49 ± 1.85	0.187
	IF	−5.24 ± 2.14	0.020^*^
HDL (mg/dl)^∧^	Control	−0.46 ± 0.24	0.062
	IF	3.04 ± 0.27	0.0001^**^
Blood glucose (mg/dl)^∧^	Control	−0.63 ± 1.63	0.703
	IF	0.62 ± 1.89	0.745

*Results are presented as mean± SEM. IF, intermittent fasting group*;

* *p < 0.05*,

** p < 0.001; parameters with

∧ *symbol represents adjustment with body weight*.

## Results

**Figure 1:**
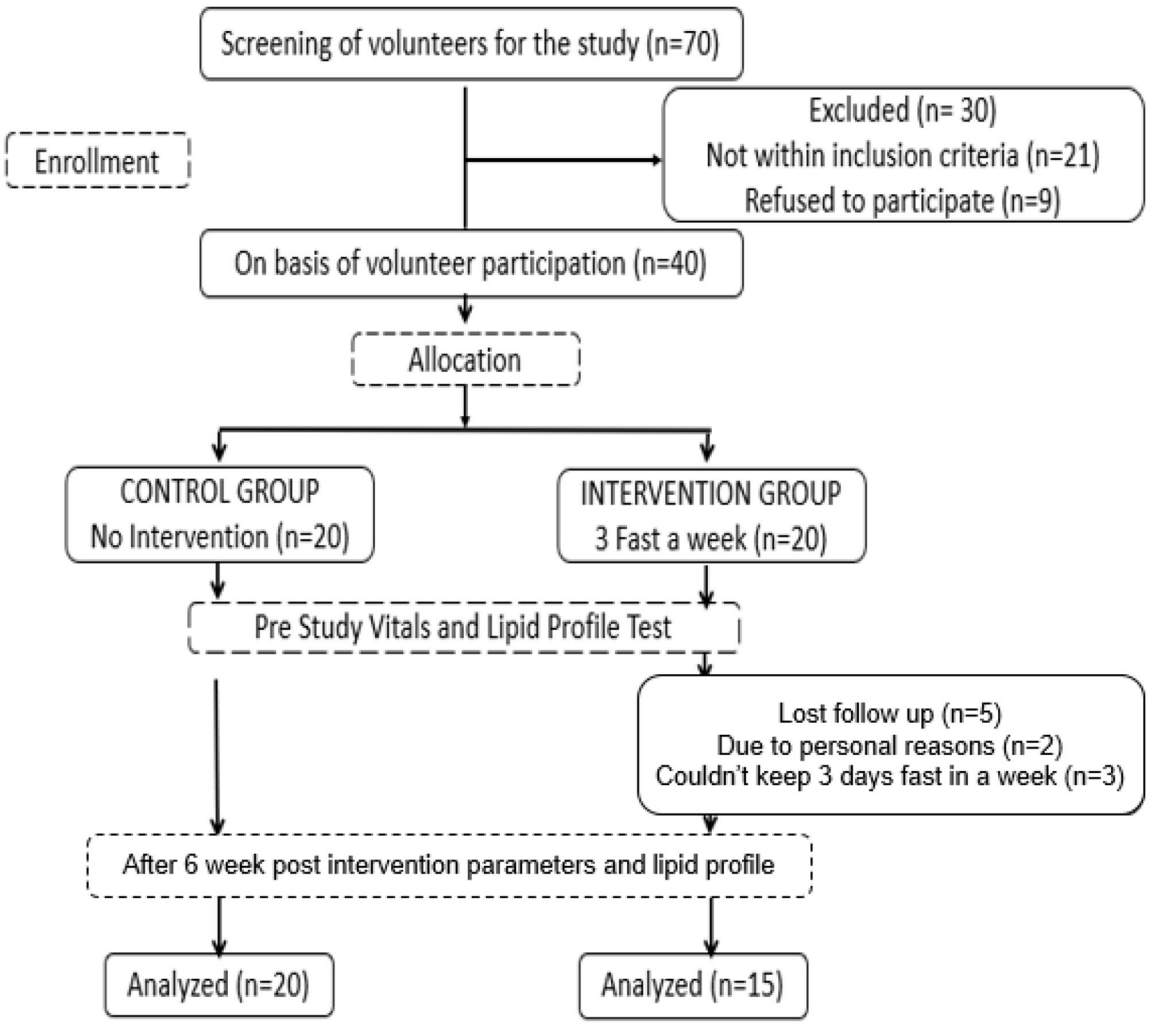
Flow chart of the study trial.

Out of 70 individuals, 40 fulfilled the inclusion criteria and were enrolled in the study – 20 in each group. Thirty-five participants (87.5%) completed the study. Five dropouts from the intervention group were due to personal reasons or inability to comply with the fasting regime. Figure 1 summarized the flow of participants through the study.

The baseline description of participants including age, gender, blood pressure, BMI level and details of their existing medical condition is represented in Table 1. The detailed questionnaire regarding eating routines and physical activity at baseline level and after post study showed no difference, all the participants followed their same daily routines as advised.

Table 2 summarize the changes in parameters at baseline and post 6 weeks study. Body measurements including body weight and BMI showed significant interaction effects (*p* = 0.0001) and time effects (*p* = 0.0001) while waist circumference showed significant interaction effect (*p* = 0.001) only. Significant interaction effects were exhibited by HDL (p=0.0001), total cholesterol (*p* = 0.033) and LDL (*p* = 0.010) with non-significant time effects. Furthermore, body fat, triglycerides and blood glucose did not show any significant interaction effects.

Table 3 shows the mean changes in body measurements, lipids, and blood glucose levels from baseline to post-treatment for the control and intervention groups and the results of *post-hoc* analyses of within-group change. The IF group had significant reductions in body weight (−3.10 ± 0.19 kg; *p* = 0.0001), BMI (−0.98 ± 0.13 kg/m^2^; *p* = 0.0001) and waist circumference (−0.98 ± 0.18 cm; *p* = 0.0001). The mean differences for IF group were also significant for total cholesterol (−16.08 ± 4.53 mg/dl; *p* = 0.001), HDL (3.04 ± 0.27 mg/dl (*p* = 0.0001), LDL (−5.24 ± 2.14 mg/dl; *p* = 0.020) and triglycerides (−12.82 ± 4.57 mg/dl; *p* = 0.008). There were no significant changes for any of the parameters for the control group. However, it should be noted that the between-group difference in change did not reach statistical significance for triglycerides.

**Figure 2:**
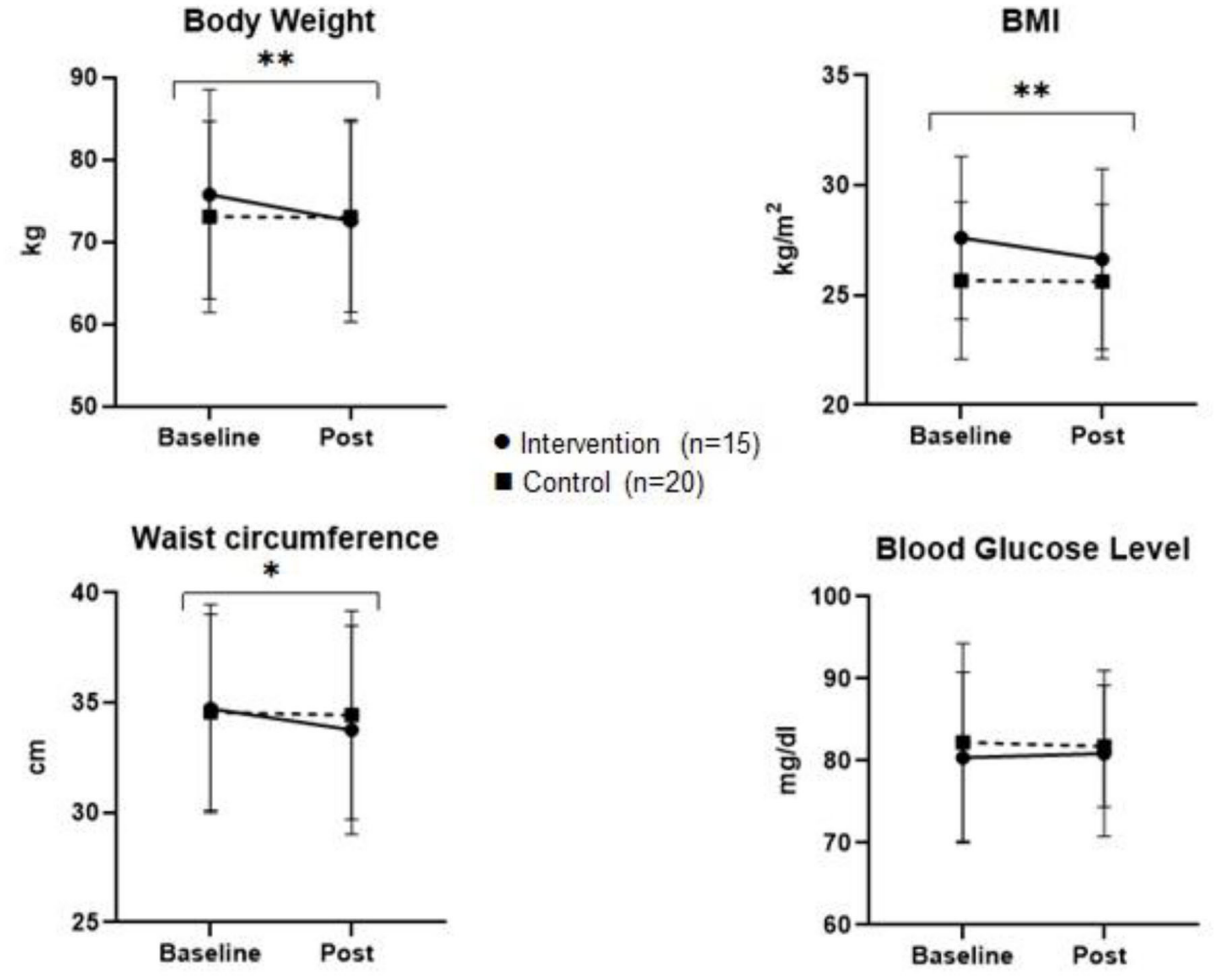
Multiplot figure of body measurements and blood glucose level of control and intervention group at baseline and post study. *p < 0.05, **p < 0.001.

**Figure 3:**
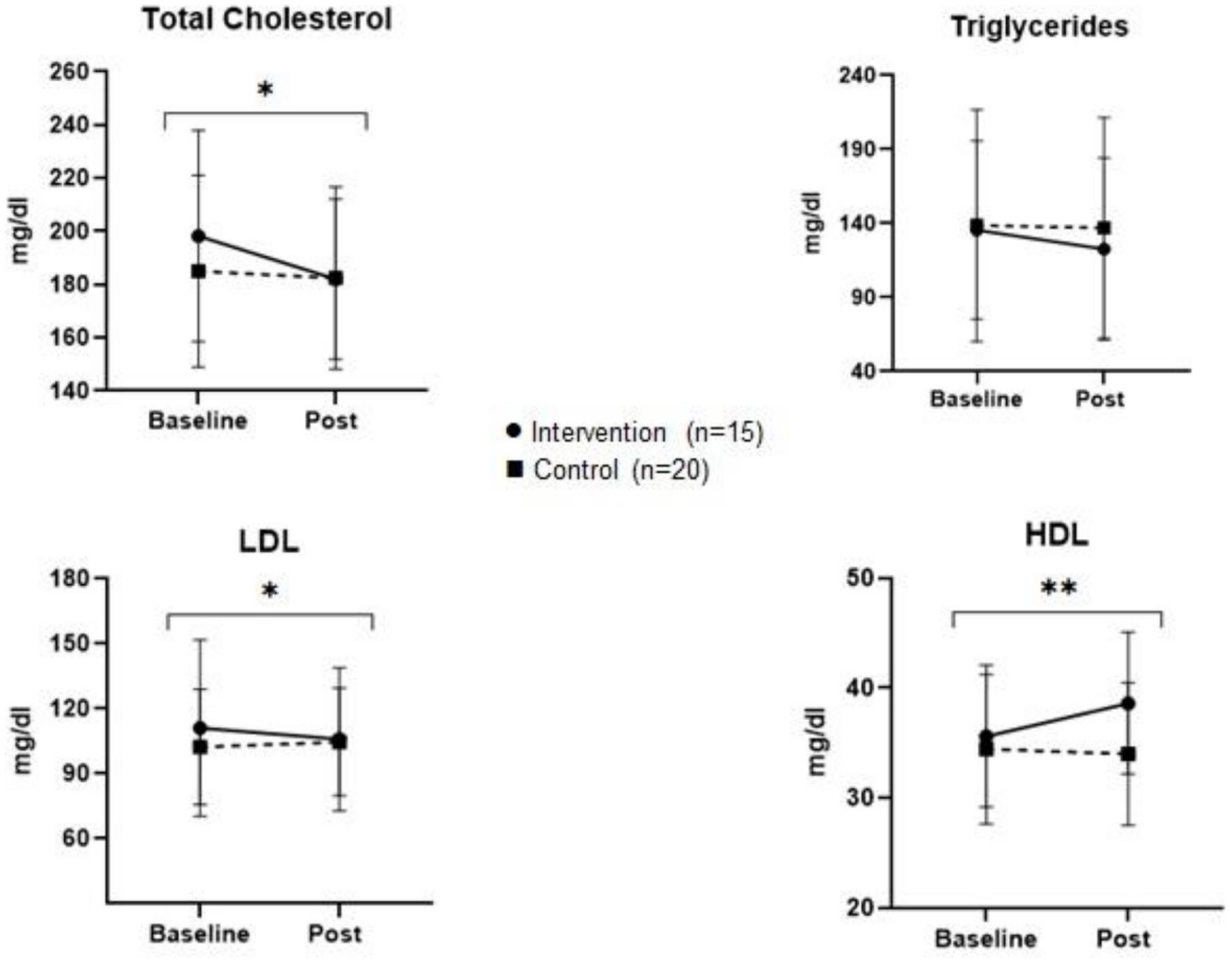
Multiplot figure of lipid levels of control and intervention group at baseline and post study. *p < 0.05, **p < 0.001.

Figures 2, 3 represent the comparison of changes in body measurements, lipid and blood glucose levels of control and intervention groups at baseline and post intervention with significance level of interaction effect.

## Discussion

The study suggests that IF has the potential of improving the lipid profile and reducing body weight and waist circumference. These results are in line with other studies showing that different types of IF, including Ramadan fasting and alternative day fasting, reduce body weight and lipid levels (17, 19). Studies combining IF with physical activity (11) and comparing different types of IF (12) also suggest that IF can be an effective lifestyle modification for reducing the risks of cardiovascular diseases. However, most of the IF clinical trials in the literature were conducted for short periods of time and large scale randomized controlled trials with longer duration and follow-ups are not available. Long term studies should be conducted to validate their effectiveness and safety.

Santos et al. have compiled data from different trials and concluded that different types of IF can increase HDL by 1–14 mg/dl, decrease LDL by 1–47 mg/ dl, decrease TC by 5–88 mg/dl and decrease TG by 3–64 mg/dl (18). As compared to the other types of IF, our method appears safe, effective and can be adopted in daily life, without any additional financial or physical burden. Individuals can incorporate IF into their lifestyles without worrying about any extra efforts to prepare low calorie meals. The 12-h fast might be maintained by an early breakfast and having dinner at an appropriate time, which works for weekdays and weekends. However, it might be difficult for people working late nights or having an active social life with frequent dining out routines. This was also observed in the current study; 5 people dropped out from the study due to their hectic and busy schedule and could not maintain fasting period for 3 day/week.

Previously conducted trials have mentioned that intermittent fasting of 12–36 h results in a metabolic switch (20) leading to a break down of triglycerides into fatty acids and glycerol and conversion of fatty acids to ketone bodies in the liver (21). During fasting, fatty acids and ketone bodies provide energy to cells and tissues (22). Studies suggested that molecule modulation in the liver leads to expression of PPARa and PGC-1a that increases fatty acid oxidation and apoA production leading to increased HDL levels, whereas apoB decreases which causes decreased hepatic triglycerides and LDL levels (23, 24). Shibata and colleagues worked on SREPB-2, Sterol regulatory element-binding protein in mice, and suggested that intermittent fasting can lead to reduction in cholesterol by regulating SREPB-2 (25).

The main limitations of this study included non-randomization of the study population. Moderate to severe dyslipidemic patients were not included in the study. Other major limitation was the drop out of five participants from the intervention group of the study which may have inflated the size of the results. It was a single centered and small-scale study lacking data on food intake and record of caloric intake.

## Conclusion

Restriction of food intake for ~12-h/day for 3 days/week leads to weight reduction and improvement in lipid profile, particularly HDL, which can reduce the risk of cardiovascular diseases. Future studies including randomized controlled trials with more diet control, longer follow ups and individuals with cardiovascular diseases and type 2 diabetes mellitus are warranted to validate these findings.

## Data Availability Statement

The raw data supporting the conclusions of this article will be made available by the authors, without undue reservation.

## Ethics Statement

The studies involving human participants were reviewed and approved by Ethical Review Committee of Aga Khan University. The patients/participants provided their written informed consent to participate in this study.

## Author Contributions

NA conceived the idea, designed and conducted the trial, provided the funding support, and supervised the study. JF conducted the study, managed the project and participants, and drafted the manuscript. HS, BK, and AHL helped in conducting the clinical trial, data interpretation, and manuscript review. HJ reviewed and revised the draft of the manuscript. FP performed the statistical analysis. SAM provided intellectual input and resources for performing some analysis. All authors contributed to the article and approved the submitted version.

## Conflict of Interest

The authors declare that the research was conducted in the absence of any commercial or financial relationships that could be construed as a potential conflict of interest.
